# Potential Fungi Isolated From Anti-biodegradable Chinese Medicine Residue to Degrade Lignocellulose

**DOI:** 10.3389/fmicb.2022.877884

**Published:** 2022-05-10

**Authors:** Min Cheng, Nalin N. Wijayawardene, Itthayakorn Promputtha, Ronald P. de Vries, Yongzhe Lan, Gang Luo, Meizhu Wang, Qirui Li, Xinyao Guo, Feng Wang, Yanxia Liu, Yingqian Kang

**Affiliations:** ^1^Key Laboratory of Medical Microbiology and Parasitology and Key Laboratory of Environmental Pollution Monitoring and Disease Control, Ministry of Education, School of Basic Medical Sciences, Guizhou Medical University, Guiyang, China; ^2^Center for Yunnan Plateau Biological Resources Protection and Utilization, College of Biological Resource and Food Engineering, Qujing Normal University, Qujing, China; ^3^Section of Genetics, Institute for Research and Development in Health and Social Care, Battaramulla, Sri Lanka; ^4^Department of Biology, Faculty of Science, Chiang Mai University, Chiang Mai, Thailand; ^5^Faculty of Science, Environmental Science Research Center, Chiang Mai University, Chiang Mai, Thailand; ^6^Fungal Physiology, Westerdijk Fungal Biodiversity Institute and Fungal Molecular Physiology, Utrecht University, Utrecht, Netherlands; ^7^Guizhou Provincial Academician Workstation of Microbiology and Health, Guizhou Academy of Tobacco Science, Guiyang, China

**Keywords:** CAZymes, filamentous fungi, fungal diversity, hydrolysis, ITS, synergistic fungal combinations

## Abstract

Traditional Chinese medicine is one of the ancient medicines which is popular in Asian countries, among which the residue produced by the use of anti-biodegradables is endless, and causes significant adverse impacts on the environment. However, the high acidity of anti-biodegradable residues and some special biological activities make it difficult for microorganisms to survive, resulting in a very low degradation rate of lignocellulose in naturally stacked residues, which directly impedes the degradation of residues. We aimed to identify the fungal strains that efficiently biodegrade anti-biodegradable residue and see the possibility to improve the biodegradation of it and other agricultural wastes by co-cultivating these fungi. We isolated 302 fungal strains from anti-biodegradable residue to test hydrolysis ability. Finally, we found *Coniochaeta* sp., *Fomitopsis* sp., *Nemania* sp., *Talaromyces* sp., *Phaeophlebiopsis* sp. which inhabit the anti-biodegradable residues are capable of producing higher concentrations of extracellular enzymes. Synergistic fungal combinations (*viz*., *Fomitopsis* sp. + *Phaeophlebiopsis* sp.; *Talaromyces* sp. + *Coniochaeta* sp. + *Fomitopsis* sp.; *Talaromyces* sp. + *Fomitopsis* sp. + *Piloderma* sp. and *Talaromyces* sp. + *Nemania* sp. + *Piloderma* sp.) have better overall degradation effect on lignocellulose. Therefore, these fungi and their combinations have strong potential to be further developed for bioremediation and biological enzyme industrial production.

## Introduction

Biomass is the fourth largest energy source in parallel with wind energy, solar energy, nuclear energy, and other new energy sources. This is considered the main alternative energy source today (Sherwood, 2020). It includes lignocellulose in the waste generated by plants, animals, and agricultural production (Zeng et al., 2013). Lignocelluloses are composed of three macromolecules: cellulose, hemicellulose, and lignin (Ni and Tokuda, 2013), which constitute an anti-hydrolysis barrier and make it difficult to be hydrolyzed (Ojha et al., 2015; Barros et al., 2016). Among them, cellulose and hemicellulose can be converted into glucose after hydrolysis. Xylose and other fermentable sugars (monosaccharides) can be further used to produce biofuels (Tan et al., 2014; Taha et al., 2016).

It has been widely studied that the application of traditional industrial and agricultural biomass, and recognized by filamentous fungi fermentation method to produce extracellular enzymes for degradation of lignocellulose has a significant effect (Lange, 2017; Kwak et al., 2019). In addition, enzymes produced by filamentous fungi have been found in nature that can be used for bioremediation, such as the ligninolytic enzyme system, which can purify heavy metal dyes in sewage, and depolymerize chlorophenols, polycyclic aromatic hydrocarbons (PAHs), organophosphorus, and phenols (Falade et al., 2017). Currently, the enzyme-producing fungi include *Trichoderma reesii*, wood/white-rotting fungi, and *Aspergillus* (Namnuch et al., 2020; Silva et al., 2020; Zhuo and Fan, 2021), which are mostly isolated from agricultural wastes such as corn straw, bagasse, wheat straw, rice husk, animal feces and some terrestrial plants (Falade et al., 2017).

However, there are also quite a number of specialized biomasses in the world, which are difficult to degraded via ordinary exogenous microorganisms due to their various characteristics, such as some extreme conditions of medical waste biomass (Hutchins et al., 2019). Isolation and screening of filamentous fungi with strong survival ability from their own accumulation environment has been increasing, and many successful cases have been reported recently. For example, Ming et al. (Ming et al., 2019) screened out several enzyme-producing filamentous fungi with strong acid tolerance from liquor grains with high acidity.

Traditional Chinese Medicine (TCM) is one of the ancient medicines which influences China and other Southeast Asian countries. The States Environment Protection Agency (SEPA; States Environment Protection Agency [SEPA], 1997) has mentioned that 11,146 species of different herbs and plants are used in TCM of which 492 species are cultivated and the remaining 10,654 species are wild plants. However, with the population growth, the demand for TCM has increased, and TCM and its residues are causing different adverse impacts on the environment (e.g., over-exploitation of plant materials, soil degradation and erosion, chemical pollution in agricultural lands). From a conservative estimate, the Chinese pharmaceutical industry of TCM can produce 60–70 million tons of residues every year, among which the TCMR (Traditional Chinese Medicine Residues) with strong anti-microbial action residues constitutes a significant proportion (Lu and Li, 2021). At the same time, such pharmaceutical residues are often accompanied by extremely low pH values, which makes it difficult for ordinary microorganisms to survive, leading to a low degradation rate of natural stacking of TCMR and irreversible damage to the environment. However, it is also a kind of agricultural biomass, containing high lignocellulose (Čater et al., 2015; Zhang and Sun, 2018; Zhu and Sun, 2018). If used correctly, it has the potential to not only reduce the pollution created by the particular environment but also to find a way for the development of biomass energy.

Meanwhile, the structure of lignocellulose is complicated and different fungi have limitations in enzyme production. Common single fungi cannot achieve complete degradation of lignocellulose (Karuppiah et al., 2021). Research shows that different microorganisms in the microbial community operate synergistically through the secretion of a variety of biocatalysts, in order to achieve comprehensive enzyme production to degrade lignocellulose (Silva et al., 2021). Therefore, we can use the method of artificial screening and mixing, fungi producing different enzymes in the same medium for co-culture to improve the degradation rate of lignocellulose.

In order to solve these problems, we report the isolation of fungi from potent anti-biodegradable TCMR, explore new lignocellulose-degrading enzymes production strains, and by using the selected strain of hydrolysis ability to build synergistic fungal combinations (co-cultivation), realize the degradation of lignocellulose. The results here would be helpful to bioremediation and develop better enzyme producers for lignocellulosic biorefinery.

## Materials and Methods

### Materials

Fungal strains were directly isolated by the spread plate method from anti-biodegradable residue which was piled up for 2–5 years at Guizhou Shuangsheng Company (Guiyang, China). Glycine, 2,2′-azino-bis (3-ethylbenzothiazoline-6-sulphonic acid; ABTS), Avicel, Briffon-Robinson 1buffer (pH 4.5), Birchwood, *p*NP-β-D-glucopyranose (*p*NP-BGL), *p*NP-β-D-xylopyranose (pNP-BXL) and other chemicals were purchased from Sigma-Aldrich to determine enzymatic activity; Carboxymethyl cellulose and xylan for solid media were purchased from Hongdaer Biotechnology Company (Guiyang, China) for preliminary screening of fungi.

### Analysis of Carbohydrate Composition and Lignin Content in Anti-biodegradable Residue

Fresh anti-biodegradable residue (younger than 2 months) was obtained from Guizhou Shuangsheng Company and ground in a blender (Conair Waring Pulverizer, Fisher Scientific), thereafter, 100 g was sieved through 50 mesh and was placed in a 40°C drying oven for 24 h. After grinding, carbohydrate composition was determined using high-performance liquid chromatography by Shandong Kechuang Quality Testing Company, and the lignin content was determined by a lignin assay kit (Solarbio, China) according to the instructions provided by the manufacturer.

### Isolation and Identification of Fungi

A suitable anti-biodegradable residue tissue block of 10 g was selected and washed with sterile normal saline three times, fungi were isolated by plant tissue separation (Makhuvele et al., 2017; Thi Minh Le et al., 2019), the sample was repeated three times. Genomic DNA of the purified fungi was extracted using Ezup Column Fungal genomic DNA Extraction Kit (Qingke, Chongqing) and used as a template for internal transcribed spacer (ITS) region amplification (Bellemain et al., 2010). Prime pair ITS5 and ITS4 (Mishra et al., 2017) was used to amplify the ITS regions (Thermal Cycler PCR instrument BIO-RAD T100, Japan). The PCR products were sequenced and used for the identification of fungi by nucleotide BLAST (Altschul et al., 1990) against the NCBI databases. The living strains were deposited at Guizhou Medical University. Phylogenetic tree (Supplementary Figure 1) was constructed by using ITS regional sequences of 302 residue isolates and by MEGA10 using the neighbor-joining (NJ) method after multiple alignments of sequences data (Saitou and Nei, 1987). The corrected evolutionary distance was evaluated according to the p-distance model (Nei and Kumar, 2000). In order to estimate the consensus of the branching, the bootstrap resampling analysis of the phylogenetic tree was employed with 1000 replicates of the data set (Felsenstein, 1985). According to the results of phylogenetic analysis, the pie chart was constructed with Adobe Illustrator CS5 software and Office.

### Preliminary Screening of Fungi on Special Media

The fungi were pre-grown on a PDA agar medium at 25°C for 3–7 days, then mycelium was transferred to three different sole carbon source media. (1) carboxymethyl cellulose Congo red agar medium (CMC-red): 1% sodium CMC, 0.05% KH_2_PO_4_, 0.05% MgSO_4_⋅7H_2_O, 1.5% agar, 0.02% Congo red, 0.2% gelatin, pH 6.8–7.2; Gupta et al., 2012; Fen et al., 2014; Wu et al., 2016); (2) xylan Congo red agar medium (Xyl-red): 0.5% xylan, 0.01% Congo red, 0.1% MgSO_4_⋅7H_2_O, 0.5% yeast extract, 0.1% KH_2_PO_4_, 1.5% agar, 0.1% (NH_4_)_2_SO_4_, 0.5% NaCl, pH 6.5–7; Liu et al., 2010), and (3) potato dextrose agar containing 0.01% aniline blue (PDA-blue), pH 6–7 (Wang H. et al., 2009). The ratio of the hydrolytic circle (D) to fungal colony diameter (d) was measured after 5 days of constant temperature incubation. Analysis of hydrolytic efficacy was performed by division of the diameter of mycelium growth by that of the clearance zone.

### Re-screening of Fungi in Liquid Culture Medium of Anti-biodegradable Residue

Fungi with high activity were cultured on a PDA plate to produce mycelium and spores. The culture medium with fungus was then placed in a sterile beaker, and spores and mycelia were separated by Tween80. Adding 2 × 10^6^ spores (Li et al., 2016) of each fungus into minimal medium (MM; composed of 4 g/L glucose and 10 g/L peptone), after shaking for 3–7 days at 28°C and a rotational speed of 30× *g*, mycelia were filtered through a 200-mesh (10 cm diameter) sieve and collected, and fully washed with sterile water. Mycelia weighing 2 g (wet weight) was transferred into a 50 ml culture medium with anti-biodegradable residue (the residue was filtered through a 50-mesh sieve) as the only carbon source (anti-biodegradable residue 3 g, Peptone 0.5 g, Yeast powder 0.25 g, NaCl 0.25 g, KH_2_PO_4_ 0.05 g, MgSO_4_⋅7H_2_O 0.025 g, (NH_4_)_2_SO_4_ 0.25 g, CuSO_4_ 0.0003 g; Wang J. F. et al., 2009; Cheng et al., 2012; Zhang et al., 2015) at 28°C, 30× *g*, 2 mL supernatant was taken at the same time on d 3, 5, 7, 9, 11, centrifuged at 4°C, 21,100× *g* for 10 min, and the clarified supernatant was taken and stored in −80°C for use.

### Construction of Synergistic Fungal Combinations (Co-cultivation)

The single filamentous fungi, which can degrade cellulose hemicellulose lignin effectively, were paired by the permutation group (Ming et al., 2019) method, in order to construct fungal combinations that can degrade lignocellulose comprehensively. The specific method is to use a sterile knife to cut the thriving fungal culture medium into 5 mm square pieces, and transfer them to a new PDA culture medium. Each piece of fungus is separated by about 1 cm. Then, the medium is sealed in a 28°C medium for 3–7 days to observe the ability of fungi to grow in co-cultivation conditions.

### Enzyme Activity Assay

β-glucosidase and β-xylosidase activities were measured with the substrates para-nitrophenyl β-glucoside (*p*NP-BGL) and para-nitrophenyl β-xyloside (*p*NP-BXL), respectively. The reaction contained 40 μl culture supernatant, 10 μl 0.1% *p*NP-BGL or *p*NP-BXL, and 50 μl 100 mM sodium acetate, pH 5.0. The reactions were performed overnight (∼16 h) at 25°C followed by the addition of 100 μl 250 mM Na_2_CO_3_ prior to measurement of the absorbance at 405 nm using a microtiter plate reader (biotek epoch2, US). Para-nitrophenol (0–100 μM) was used as the standard (Patyshakuliyeva et al., 2016). One unit was defined as the amount of enzyme releasing 1 μmol *p*NP from *p*NP-BGL or *p*NP-BXL under the assay condition.

Cellulase and xylanase activities were measured toward avicel cellulose and birchwood xylan, respectively. The reaction contained 20 μl culture supernatant and 180 μl 1% avicel or birch xylan in 50 mM sodium acetate, pH 5.0, and was incubated overnight (∼16 h) at 25°C. Mixing 100 μl of the reaction mixture with 150 μl 3,5-dinitrosalicylic acid solution (1% 3,5-dinitrosalicylic acid, 0.2% phenol, 0.05% Na_2_SO_3_, and 1% NaOH), incubated at 95°C, 30 min and cooled on ice prior to measurement of the absorbance at 560 nm using a microtiter plate reader (Miller, 1959). 2–20 mM D-glucose or D-xylose was used as standard (Peciulyte et al., 2017).

Peroxidase and laccase activities were measured toward 2,2′-azinobis (3-ethylbenzthiazoline-6-sulfonic acid; ABTS) with or without supplement of hydrogen peroxide, respectively. The peroxidase reaction contained 20 μl culture supernatant, 20 μl 140 μM ABTS, 10 μl 3% H_2_O_2_, 25 μl 400 mM Britton–Robinson buffer, pH 4.5, and 125 μl ddH_2_O. Detection of initial rates of oxidation was performed at 440 nm with a 2 min interval up to 60 min, at 25°C using a microtiter plate reader. The laccase reaction contained 20 μl culture supernatant, 20 μl 140 μM ABTS, 20 μl 500 mM glycine-HCl, pH 3.0, and 140 μl ddH_2_O. The initial rates of hydrolysis were determined by measuring absorbance at 440 nm in a 2 min interval for 30 min, at 25°C. Laccase and peroxidase activities were calculated based on Lambert-Beer law, where the extinction coefficient of 3.6× 10^4^ M^–1^ cm^–1^ was used as mentioned in Childs and Bardsley (1975) and Srinivasan et al. (1995).

### Hydrolytic Weight Loss Ability Test

The selected co-cultivation was incubated at 28°C for 15 days, and the actual hydrolysis capacity of the residue was tested. The residue was taken from the liquid medium and thoroughly dried before weighing to determine the total weight loss rate of the residue as well as the degradation rates of cellulose, hemicellulose, and lignin. Each strain was repeated three times, and the residue liquid medium without inoculation was used as a negative control. The negative control M_0_ was used to calculate the hydrolysis rate of residue and cellulose, instead of residue weight before inoculation of fungi M_1_, in order to eliminate the natural loss of residue powder placed in the solution during the experiment and ensure the strictest of the experiment. The formula for calculating the total weight loss rate of drug residue hydrolysis is (M_0_-M_2_)/M_0_, where M_0_ represents the dry weight of drug residue after 15 days of culture in a negative control group under the same conditions, and M_2_ represents the weight of drug residue after hydrolysis (Ming et al., 2017). Hemicellulose and lignin content in the hydrolyzed residue was detected by the content detection Kit (Solarbio BC4445, Solarbio BC4205) and cellulose content detection kit (ZCIBIO ZC-S0876). The specific operation method is described in the instruction, three biological replicates were performed.

## Results

### Identification of Strains

The ITS sequences were used to identify the strains from mega blast results in GenBank. In this preliminary study, we only intended to check whether there are fungal taxa that are inhabiting TCM residues. In Supplementary Table 1, we provide results that were generated in the mega blast. It provides the closest hits which are similar to our selected strains.

### Determination of the Composition of Anti-biodegradable Residue

The determination of glycosylated components in the residue showed that glucose, accounting for 68.32%, glucose is the reducing sugar of cellulose, indicating that the sample contains a lot of cellulose. Plus, there were xylose, arabinose, mannose, galactose, and other five-carbon sugars, accounting for about 30% (Table 1), and the analysis of lignin content in the residue showed that there was 13.66% lignin. The lignin content test suggests the anti-biodegradable residue contains a moderate amount of lignin in liquid samples. In addition, 50 ml of 6% residue is pH 4.9.

**TABLE 1:** Table of carbohydrate composition of anti-biodegradable residue.

Carbohydrate composition	mg/kg	Accounted for (%)
Mannose	3694.24	0.7
Ribose	631.99	0.12
Rhamnose	3626.84	0.69
Glucuronic acid	1783.52	0.34
Galacturonic acid	11096.61	2.13
Glucose	355769.68	68.32
Galactosum	18197.96	3.49
Xylose	84775.66	16.28
Arabinose	39887.86	7.66
Fucose	1240.14	0.24

### Biodiversity and Phylogenetic Analysis of Isolated Filamentous Fungi

**FIGURE 1:**
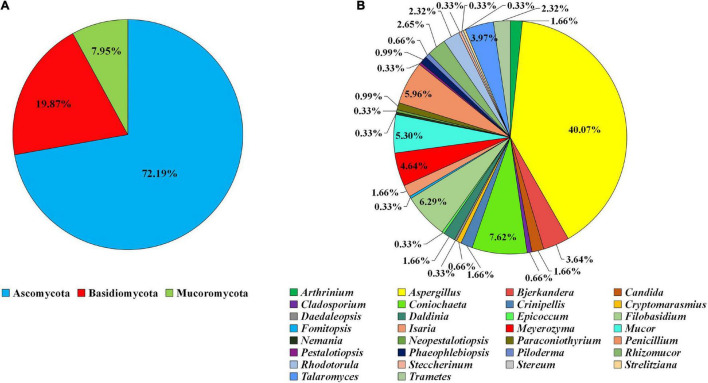
Composition of the filamentous fungal strains isolated from anti-biodegradable residue. **(A)** The classification was shown at the phyla level. **(B)** Generic level.

A total of 302 fungi were isolated and labeled as ZYJHYZ01-ZYJHYZ302 (Supplementary Table 2). Phylogenetic analysis showed that they belong to 30 genera in 3 phyla. Among the isolates, 72.19% of strains (218 isolates of 16 genera) were identified as Ascomycota, while 19.87% of strains (60 isolates of 12 genera) were identified as Basidiomycota and 7.95% strains (24 isolates of two genera) were identified as Mucoromycota (Figure 1A). At the genus level, 40.07% of isolates (*n* = 121) belong to *Aspergillus*, 7.62% to *Coniochaeta* (*n* = 23), 6.29% to *Filobasidium* (*n* = 19), 5.96% to *Penicillium* (*n* = 18), and 5.30% to *Mucor* (*n* = 16; Figure 1B).

### A Single Polymer Was Used as the Sole Carbon Source in Solid Medium With Indicator

Three-hundred and two (302) isolates were screened by inoculating onto a solid medium with a single polymer (CMC, xylan, or glucose) as the sole carbon source and indicator added: CMC-red, PDA-blue, Xyl-red, 25°C, the ratio of the hydrolytic circle (D) to fungal colony diameter (d) was measured after 5 days of constant temperature incubation. From this analysis, we were able to find that 35 strains produced transparent circles while 24 strains showed a ratio greater than or equal to 2 (Supplementary Table 3).

**FIGURE 2:**
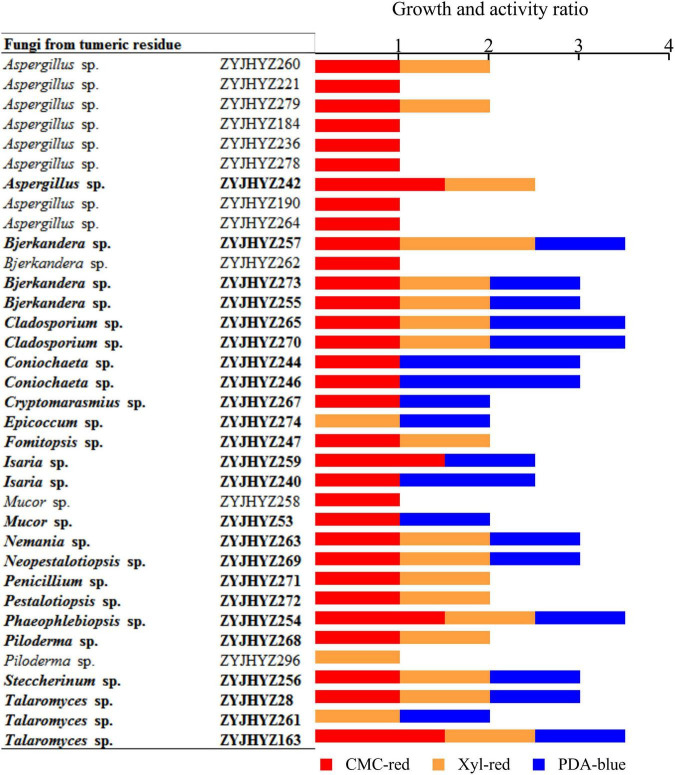
Growth and activity ratio of the selected fungi grown on solid media. The media contains 1% cellulose (CMC-red), 0.5% xylan (Xyl-red) or 0.01% aniline blue (PDA-blue). Fungi in bold showed > 2.0 growth ratio and were chosen for cultivation in liquid media. The growth and activity ratio was estimated by hydrolytic diameter/colony diameter after 5 days.

Among them, the D/d ratio of ZYJHYZ163, ZYJHYZ254, ZYJHYZ257, ZYJHYZ265, and ZYJHYZ270 were greater than other strains. According to the mega blast results, *Aspergillus fumigatus* (ZYJHYZ279) and *Aspergillus flavus* (ZYJHYZ260) were indicated as harmful to human and animal health, thus, further experimental studies were abandoned. Therefore, fungi with a total score of 2 were preliminarily screened through three kinds of solid media for the next experiment, and a total of 24 strains were identified: ZYJHYZ257, ZYJHYZ259, ZYJHYZ261, ZYJHYZ263, ZYJHYZ265, ZYJHYZ267, ZYJHYZ268, ZYJHYZ269, ZYJHYZ270, ZYJHYZ271, ZYJHYZ272, ZYJHYZ273, ZYJHYZ274, ZYJHYZ53, ZYJHYZ163, ZYJHYZ244, ZYJHYZ242, ZYJHYZ28, ZYJHYZ246, ZYJHYZ254, ZYJHYZ255, ZYJHYZ240, ZYJHYZ247, and ZYJHYZ256 (Figure 2).

### Comparison of Lignocellulose Degrading Enzyme Capacity of Single-Cultured Fungi

**FIGURE 3:**
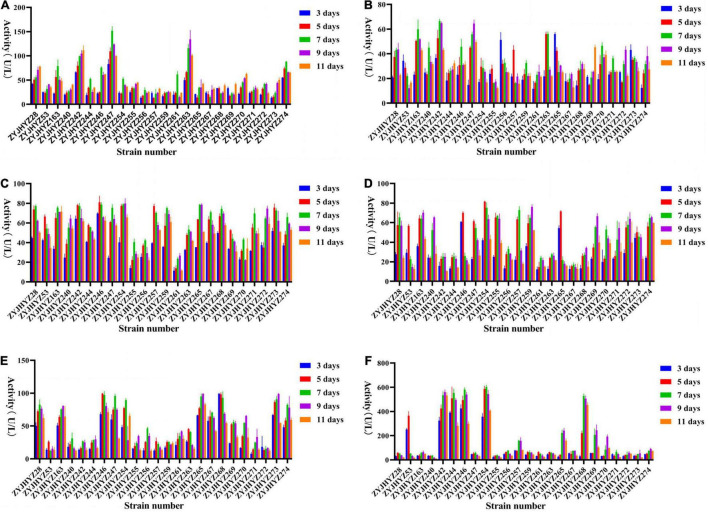
Extracellular enzyme activities of selected fungi for pre-screening. **(A)** β-glucosidase, **(B)** Cellulase, **(C)** β-xylosidase, **(D)** Xylanase, **(E)** Laccase, and **(F)** Peroxidase activity of culture supernatant of fungi grew in 6% anti-biodegradable residue (in water) as the sole carbon source. The culture supernatants were collected on 3, 5, 7, 9, and 11 days after the inoculation. Three biological replicates were performed for each of the above data.

All fungi produce different levels of enzymes with different activities. Here, we screened for six different enzymes, *viz*., β-glucosidase, cellulase, β-xylosidase, xylanase, laccase, and peroxidase. **β-glucosidase** – In general, most fungi can produce higher β-glucosidase activity on days 7–9 (Figure 3A), among which *Fomitopsis* sp. (ZYJHYZ247) produces the highest β-glucosidase level on day 7 (151.68 U/L) followed by *Nemania* sp. (ZYJHYZ263) reached 134.22 U/L on day 9. **Cellulase** – However, the peak time of production was different among different fungal strains, among which *Aspergillus niger* (ZYJHYZ242) reached 66.55 U/L on day 7, and *Fomitopsis* sp. (ZYJHYZ247) reached 64.63 U/L on day 9 (Figure 3B). **β-xylosidase** – Figure 3C, showed that multiple fungi had the same enzyme production, among which the best enzyme-producing fungus was *Coniochaeta* sp. (ZYJHYZ246) and *Phaeophlebiopsis* sp. (ZYJHYZ254). *Coniochaeta* sp. (ZYJHYZ246) had the highest enzyme production at 80.97 U/L on the eighth day while *Phaeophlebiopsis* sp. (ZYJHYZ254) had the highest enzyme production at 79.92 U/L on the nineth day. **Xylanase** – In Figure 3D, *Phaeophlebiopsis* sp. (ZYJHYZ254) had the highest enzyme production, with the highest enzyme production time reaching 81.59 U/L on the 5th day, *Bjerkandera* sp. (ZYJHYZ257) and *Isaria* sp. (ZYJHYZ259) performed higher xylanase activity at day 9 and 7 with 76.14 and 72.98 U/L, respectively, in addition, *Talaromyces* sp. (ZYJHYZ163) also showed excellent and stable β-xylosidase and xylanase activity. **Laccase** – From Figure 3E, strain *Piloderma* sp. (ZYJHYZ268) reached the highest enzyme production (99.39 U/L) on the fifth day, followed by *Coniochaeta* sp. (ZYJHYZ246) and *Cladosporium* sp. (ZYJHYZ265) with 99.31 and 99.24 U/L, respectively. **Peroxidase** – From Figure 3F, including *Phaeophlebiopsis* sp. (ZYJHYZ254), *Piloderma* sp. (ZYJHYZ268), *Coniochaeta* sp. (ZYJHYZ246), *Coniochaeta* sp. (ZYJHYZ244), *Aspergillus* sp. (ZYJHYZ242) showed significantly higher enzyme activity than other fungi, with *Phaeophlebiopsis* sp. (ZYJHYZ254) having the highest enzyme activity, reaching 602.96 U/L on day 7, followed by *Coniochaeta* sp. (ZYJHYZ246) and *Aspergillus* sp. (ZYJHYZ242) with 580.42 and 561.06 U/L, respectively.

In conclusion, strains belonging to *Coniochaeta*, *Fomitopsis*, *Phanerochaete*, and *Piloderma* are capable of producing a higher amount of lignocellulose degrading enzymes compared to other strains.

### Construction of Fungal Strains and Their Co-cultivation Potential to Improve Enzymatic Degradation

**FIGURE 4:**
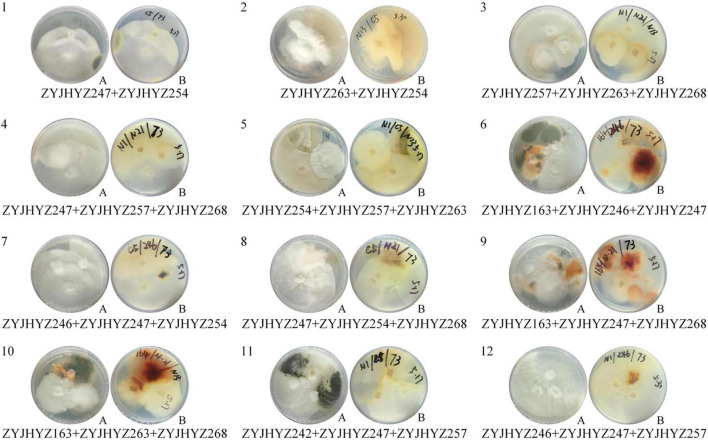
Growth of fungi in synergistic combinations (co-cultivation). 1, 2, 3, 4… is co-cultivation code; **(A)**. from above, **(B)**. from below.

**FIGURE 5:**
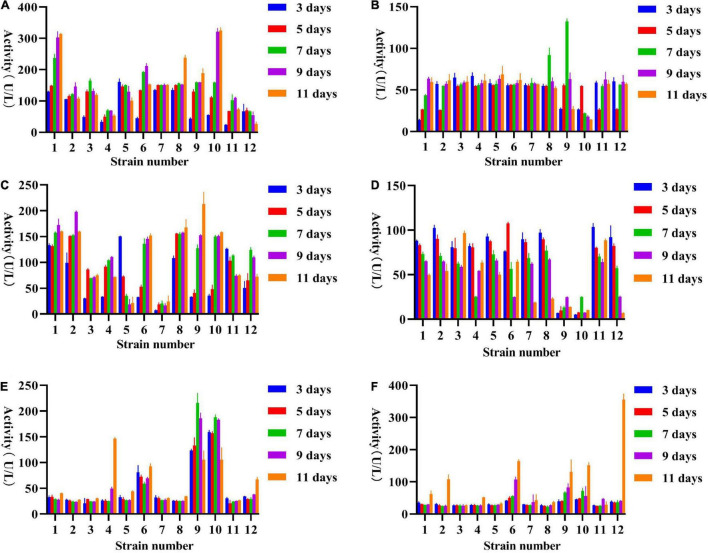
Extracellular enzyme activities of sixteen fungal co-cultivation supernatant at days 3, 5, 7, 9, and 11 after inoculation in water with 6% anti-biodegradable residue; **(A)** β-glucosidase, **(B)** Cellulase, **(C)** β-xylosidase, **(D)** Xylanase, **(E)** Laccase, and **(F)**. Peroxidase. Three biological replicates were performed for each of the above data.

Through the screening for six fungal extracellular enzymes, the first three fungal strains with the highest activity of each enzyme were selected for further co-cultivation; *Aspergillus* sp. (ZYJHYZ242), *Fomitopsis* sp. (ZYJHYZ247), and *Nemania* sp. (ZYJHYZ263) for cellulose degradation. *Talaromyces* sp. (ZYJHYZ163), *Phaeophlebiopsis* sp. (ZYJHYZ254), and *Bjerkandera* sp. (ZYJHYZ257) for hemicellulose degradation. High lignin-degrading strains numbered as *Coniochaeta* sp. (ZYJHYZ246), *Phaeophlebiopsis* sp. (ZYJHYZ254), and *Piloderma* sp. (ZYJHYZ268), the BLAST results from NCBI as Supplementary Table 3. A total of 27 fungal co-cultivation was constructed by permutation and combination of the above fungi, in order to screen out fungi that can comprehensively degrade lignocellulose. Twelve co-cultivation were growing together through the symbiosis test (Figure 4) and their enzyme activities showed in Figure 5. The other strain combinations were excluded from liquid cultivation.

### Comparison of Lignocellulose Degrading Enzyme Capacity of Co-cultivation Fungi

According to Figure 5, the activities of 5 enzymes (except for lignin peroxidase) have been greatly improved over those strains in single culture conditions. β-glucosidase – contained more than 100 U/L activities in most fungal combinations while only three single-cultured fungi were capable of production at this level. The highest β-glucosidase was produced on day 11 of incubation by fungal co-cultivation no. 1 (313.63 U/L), followed by no. 10 (325.03 U/L; Figure 5A). Cellulase – showed that multiple fungi had the same enzyme production, the cellulase activity performed highest on the seventh after inoculation by fungal combination nos. 8 and 9 with 91.86 and 132.43 U/L, respectively (Figure 5B), the highest enzyme activity of no. 9 reached 132.43 U/L, was about twice that of a single fungus. β-xylosidase – the peak of enzyme production in combinations nos. 5 and 11 was on the third day, and then decreased with time, while the other fungal combinations had an opposite trend, mostly reaching the peak around the ninth day, and β-xylosidase activity performed highest at day 11 and 9 after inoculation by fungal combination nos. 9 and 2 with 213.07 and 197.86 U/L, respectively (Figure 5C). Xylanase – most fungal combinations showed the highest xylanase activity from day 3 to day 5, among which the highest was no. 6, which reached 107.47 U/L on day 5, followed by no. 11 (103.55 U/L at day 3; Figure 5D). Laccase – from Figure 5E, the enzyme activity of no. 4, 9, and 10 were significantly higher than that of other combinations. The enzyme activity of no. 9 reached 215.46 U/L on the seventh day of inoculation, more than twice that of a single fungus, followed by no. 10 (187.69 U/L). Peroxidase – it can be seen from Figure 5F that the peak of enzyme production of almost all fungal combinations was on day 11, and the enzyme activity of fungal combination no. 12 on day 11 was at least twice that of other fungal combinations, reaching 355.65 U/L. In addition, the enzyme activity of fungal combinations no. 6, 9, and 10 are also relatively advantageous, but not as high as that of single fungi (Figure 5F).

Thus, we suggest using co-cultivation *viz*., no. 1 *Fomitopsis* sp. (ZYJHYZ247) + *Phaeophlebiopsis* sp. (ZYJHYZ254); no. 6 *Talaromyces* sp. (ZYJHYZ163) + *Coniochaeta* sp. (ZYJHYZ246) + *Fomitopsis* sp. (ZYJHYZ247); no. 9 *Talaromyces* sp. (ZYJHYZ163) + *Fomitopsis* sp. (ZYJHYZ247) + *Piloderma* sp. (ZYJHYZ268); no. 10 *Talaromyces* sp. (ZYJHYZ163) + *Nemania* sp. (ZYJHYZ263) + *Piloderma* sp. (ZYJHYZ268) to degrade the anti-biodegradable residue rather introducing them as a monoculture.

### Hydrolytic Weight Loss Ability Test of Co-cultivation

**FIGURE 6:**
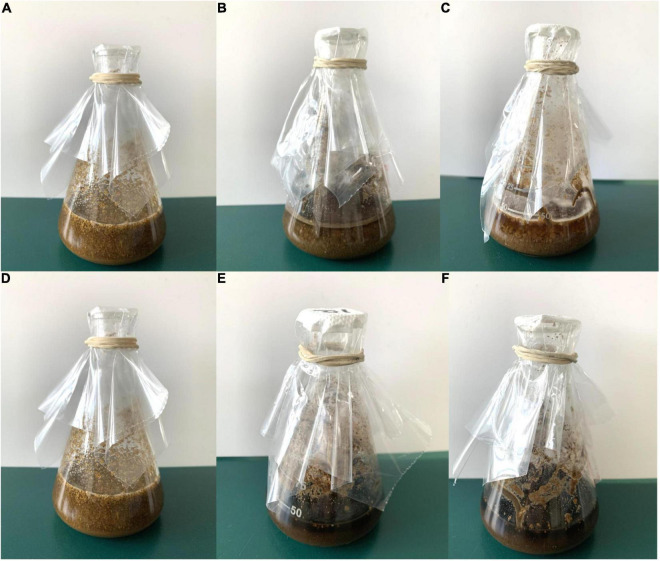
Residue after 15 days of fermentation in liquid medium. **(A)** and **(D)** Negative control, **(B)** no. 1, **(C)** no. 6, **(E)** no. 9, and **(F)** no. 10.

In the co-cultivation, fermentation broth with anti-biodegradable residue as the sole carbon source was added at a constant temperature of 30× g at 28°C for 15 days (Figure 6). Compared with the negative control, about half of the residue was left in nos. 1 and 6, and about one-third of residue was left in nos. 9 and 10. The overall weight loss rate of TCMR is shown in Table 2. According to Table 2, the weight loss rate of no. 10 hydrolysis for 15 days is the highest, reaching 60.34%. The overall weight loss data is also consistent (Figure 6). Results of lignocellulosic content are shown in Table 3. It can be seen that the weight loss rate of cellulose in no. 1 after 15 days of hydrolysis is the highest, reaching 57.46%. Nos. 9 and 10 had the strongest hydrolysis ability for hemicellulose, and the weight loss rate of no. 10 reached 38.83%. No. 9 had the strongest hydrolysis capacity of lignin, reaching 64.47%.

**TABLE 2:** Total weight loss rate of residue after 15 days of hydrolysis.

	Negative control	1	6	9	10
Initial amount of residue (g)	3	3	3	3	3
Residual amount after 15 days of fermentation (g)	2.7090	1.6625	1.8726	1.1615	1.0744
Weight-loss ratio (%)		38.63	30.87	57.12	60.34

**TABLE 3:** Lignocellulose weight loss rate after 15 days of hydrolysis.

	1	6	9	10
Weight-loss rate of cellulose (%)	57.46	29.34	36.43	51.34
Weight-loss rate of hemicellulose (%)	25.21	15.55	37.34	38.83
Weight-loss rate of lignin (%)	11.57	30.75	64.47	59.68

## Discussion

Herbal medicine plays a vital role in Asian countries and has a long history. Kloos mentioned that TCM has an average of 15% of annual growth between 2011 and 2016 (Kloos, 2017). Besides, Dang et al. (2016) reported that the value of different products of TCM is over 120 billion USD by the end of 2014 which represents 31% of the total pharmaceutical industry market in China. Hence, TCM is still in considerable demand. This also directly leads to the discharge of a large number of TCMR, long-term accumulation is currently the most adopted method, for environmental protection and economic development are great challenges. In recent years, they are often used for fermentation and composting research, due to the special properties of the TCMR (such as high acidity and strong bacteriostasis). In this study, the characteristics of the residue itself are taken as the starting point to screen out the fungi that can resist such an unusual environment, so as to solve the environmental pressure and lay a foundation for the development of bioenergy.

Three hundred and two (302) strains that belong to 30 genera have been isolated. A total of 35 strains with lignocellulosic degradation ability were obtained by single polymer solid medium screening. According to the diameter ratio, 24 strains were further screened out for 6 plant-degrading enzyme activities: β-glucosidase, cellulase, β-xylosidase, xylanase, laccase, and peroxidase. Among these six enzymes, we found that most fungi in 7–9 days can produce higher β-glucosidase and Cellulase. *Aspergillus* sp. (ZYJHYZ242) and *Fomitopsis* sp. (ZYJHYZ247) had the highest enzyme activity and the best cellulose degradation potential. *Aspergillus* is an important industrial fermentation strain that can produce cellulose (Van Munster et al., 2020; Zhao et al., 2020). *Fomitopsis* has also been repeatedly reported as a model brown rot fungus that causes destructive wood decay based on cellulase systems, producing cellulolytic enzymes and lignin decomposing enzymes (Hong et al., 2017). In the detection process of enzymes related to lignin degradation, it was found that the peak of enzyme production of most strains was 5–7 days, among which *Phaeophlebiopsis* sp. (ZYJHYZ254) had the highest peroxidase production, which reached 602.96 U/L on the seventh day, which was two to three times of the fungi with high total lignin peroxidase production reported in the current literature (Saito et al., 2018; Su et al., 2018). In addition, *Phaeophlebiopsis* sp. (ZYJHYZ254), *Piloderma* sp. (ZYJHYZ268), and *Coniochaeta* sp. (ZYJHYZ246) were also the three strains that produced the highest lignin-degrading enzymes. *Coniochaeta* was also reported to produce β-glucosidase, cellulase, hemicellulase and laccase (Mondo et al., 2019). *Piloderma* is a common ectomycorrhizal fungus (EMF) in forest soil. It is well known that EMF can produce a large number of peroxidase to degrade the complex structure containing lignin (Heinonsalo et al., 2015). Recently, Rineau et al. also found more than one laccase gene was encoded in the genome of *Piloderma* (Rineau et al., 2012). In the hemicellulase activity results, *Phaeophlebiopsis* sp. (ZYJHYZ254) had the highest xylanase production, and the highest enzyme production time was on the fifth day, and β-xylosidase production was similar among the fungi. The best enzyme-producing fungi was *Coniochaeta* sp. (ZYJHYZ246) and *Phaeophlebiopsis* sp. (ZYJHYZ254).

Nevertheless, studies have shown that a single strain cannot degrade lignocellulose completely (Karuppiah et al., 2021). Hence, it is recommended to construct co-cultivation to achieve complete degradation of lignocellulose. The main aim of the co-cultivation is to improve the overall lignocellulolytic activity, as lignocellulose is the main component in agricultural waste (Čater et al., 2015; Ming et al., 2019). However, it is not feasible to predict which strains will be able to grow together. Hence, we first grew different combinations on solid complex media (Hu et al., 2011). Strain combinations showing a separation zone or barrage line indicated incompatibility among the strains. These strain combinations were excluded from liquid cultivation. The enzyme activity of co-cultivation (12 groups) that can grow together was detected. It was found that except for lignin peroxidase, the enzyme activity of the other 5 enzymes had a significant increase, with the same amount of mycelium inoculation as that of a single strain, the effect of enzyme production of the co-cultivation was two to three times higher than that of a single strain, indicating that the co-cultivation was successfully constructed. However, it should be noted that the enzyme activity of the co-cultivation of Figure 5F was generally lower than that of the single fungi, which may be due to the interaction of various chemical substances produced by fungi, resulting in low overall enzyme activity.

To sum up, we believe that co-cultivation *viz*., no. 1 *Fomitopsis* sp. (ZYJHYZ247) + *Phaeophlebiopsis* sp. (ZYJHYZ254); no. 6 *Talaromyces* sp. (ZYJHYZ163) + *Coniochaeta* sp. (ZYJHYZ246) + *Fomitopsis* sp. (ZYJHYZ247); no. 9 *Talaromyces* sp. (ZYJHYZ163) + *Fomitopsis* sp. (ZYJHYZ247) + *Piloderma* sp. (ZYJHYZ268); no. 10 *Talaromyces* sp. (ZYJHYZ163) + *Nemania* sp. (ZYJHYZ263) + *Piloderma* sp. (ZYJHYZ268) has a better overall degradation potential of lignocellulose. Based on these results, we conclude that wood white rots fungi and other inhabiting fungi are capable of producing higher concentrations of extracellular enzymes, such as *Coniochaeta* sp. (ZYJHYZ246), *Fomitopsis* sp. (ZYJHYZ247), and *Phaeophlebiopsis* sp. (ZYJHYZ254).

Nevertheless, as this is not a taxonomy paper, we did only preliminary identification for strains. It is essential to carry out multi-gene analyses to identify cryptic species in *Aspergillus* and other prominent species which are inhabiting species complexes. Current results showed that the all isolated strains are acid-resistant, and with the extension of residue stacking time, their pH value will be lower, the diversity of fungi will gradually decline, and the nutritional type of strains will tend to be single. Therefore, it can be predicted that the longer the residue stacking time, the more likely it is to isolate the lignocellulose-degrading enzymes strain. It is generally believed that the unique high acidity and bacteriostatic environment of TCMR tend to give it special characteristics of its own microorganisms, so the special functions of isolated strains can be further discussed.

## Data Availability Statement

The original contributions presented in the study are included in the article/Supplementary Material, further inquiries can be directed to the corresponding author.

## Author Contributions

MC, RdV, YL, and XG performed the material preparation, data collection, and analysis. MC, NW, and IP wrote the first draft of the manuscript. FW, YL, GL, MW, QL, and YK advanced the manuscript revision and examination. All authors contributed to the conception and design of the study, commented on previous versions of the manuscript, and read and approved the final manuscript.

## Conflict of Interest

The authors declare that the research was conducted in the absence of any commercial or financial relationships that could be construed as a potential conflict of interest.

## Publisher’s Note

All claims expressed in this article are solely those of the authors and do not necessarily represent those of their affiliated organizations, or those of the publisher, the editors and the reviewers. Any product that may be evaluated in this article, or claim that may be made by its manufacturer, is not guaranteed or endorsed by the publisher.
