# Supplementary material for: Potential Fungi Isolated From Anti-biodegradable Chinese Medicine Residue to Degrade Lignocellulose
Source: Front Microbiol. 2022 May 10;13:877884. doi: 10.3389/fmicb.2022.877884 (PMC9127797; doi:10.3389/fmicb.2022.877884)
Supplement: Supplementary file 1 [file Data_Sheet_1.DOCX]

**
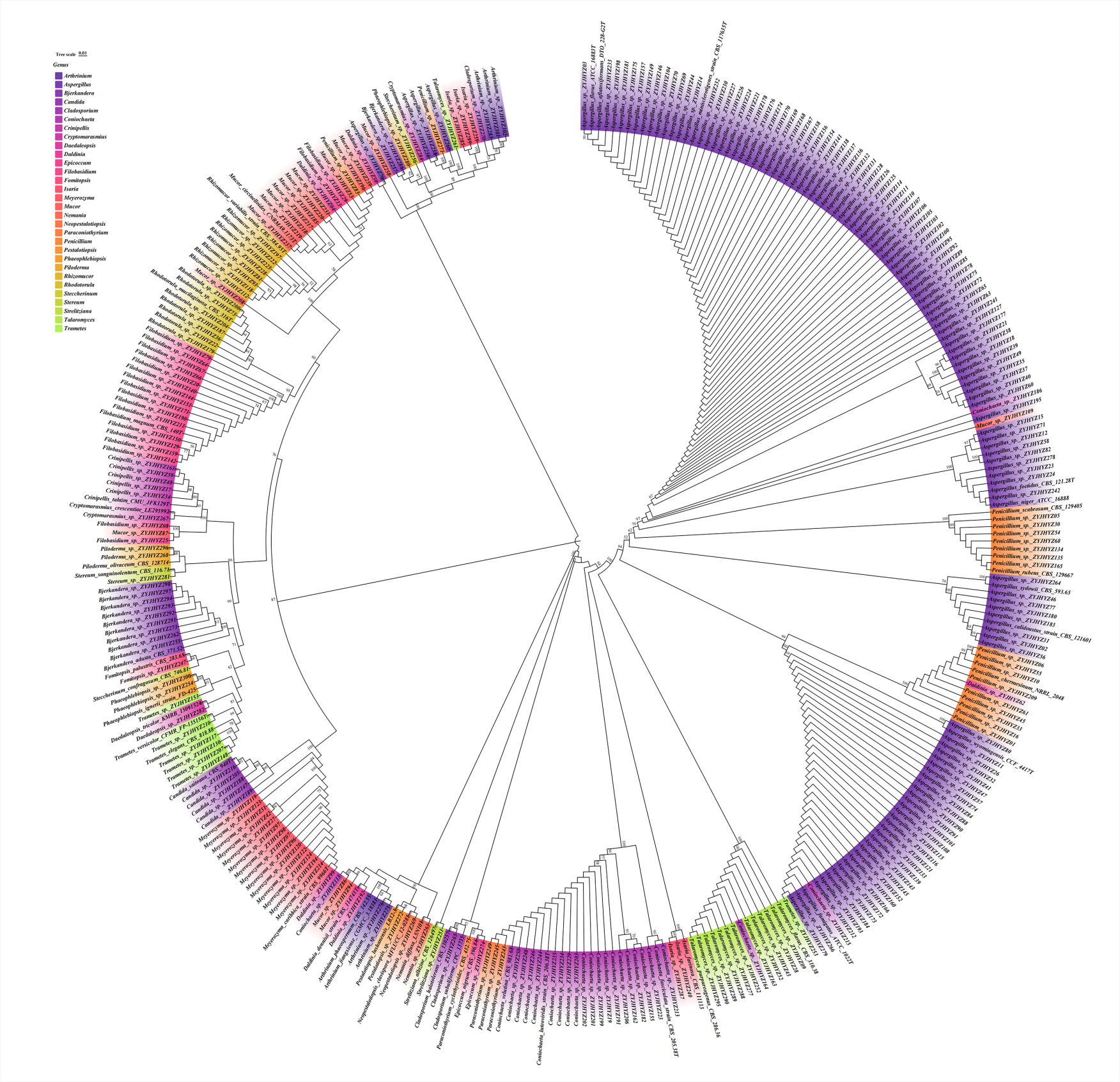
Supplementary Figure S1. Evolutionary relationships of 302 fungi strains**

The evolutionary history was inferred using the Neighbor-Joining method (Saitou and Nei, 1987). The optimal tree with the sum of branch length = 7.08619568 is shown. The percentage of replicate trees in which the associated taxa clustered together in the bootstrap test (1000 replicates) are shown next to the branches (Felsenstein, 1985). The tree is drawn to scale, with branch lengths in the same units as those of the evolutionary distances used to infer the phylogenetic tree. The evolutionary distances were computed using the p-distance method (Nei and Kummar, 2000) and are in the units of the number of base differences per site. The analysis involved 348 nucleotide sequences. All positions with less than 50% site coverage were eliminated. That is, fewer than 50% alignment gaps, missing data, and ambiguous bases were allowed at any position. There were a total of 438 positions in the final dataset. Evolutionary analyses were conducted in MEGA10 and display and annotation by online tool iTOL (http://itol.embl.de) and CHIplot (https://www.chiplot.online/).

**References**

Felsenstein., J. (1985). Confidence limits on phylogenies: an approach using the bootstrap. *Evolution*. 39, 783-791. doi: 10.1111/j.1558-5646.1985.tb00420.x

Nei., M. and Kumar., S. (2000). Molecular evolution and phylogenetics. Oxford University Press. New York

Saitou., N., and Nei., M. (1987). The neighbor-joining method: a new method for reconstructing phylogenetic trees. *Mol. Biol. Evol*. 4, 406-425. doi:10.1093/oxfordjournals.molbev.a040454
