# Supplementary material for: Potential Fungi Isolated From Anti-biodegradable Chinese Medicine Residue to Degrade Lignocellulose
Source: Front Microbiol. 2022 May 10;13:877884. doi: 10.3389/fmicb.2022.877884 (PMC9127797; doi:10.3389/fmicb.2022.877884)
Supplement: Supplementary file 2 [file Table_1.DOC]

**Table S1 Isolated strain number, species name and its ITS accession number**

| **Taxon** | **Strain number** | **ITS accession no.** |
| --- | --- | --- |
| *Arthrinium* sp. | ZYJHYZ276 | MW659148 |
| *Arthrinium* sp. | ZYJHYZ275 | MW659147 |
| *Arthrinium* sp. | ZYJHYZ283 | MW659155 |
| *Arthrinium* sp. | ZYJHYZ301 | MZ321998 |
| *Arthrinium* sp. | ZYJHYZ302 | MZ321999 |
| *Aspergillus* sp. | ZYJHYZ02 | MT312865 |
| *Aspergillus* sp. | ZYJHYZ31 | MT312876 |
| *Aspergillus* sp. | ZYJHYZ46 | MT312885 |
| *Aspergillus* sp. | ZYJHYZ56 | MT312888 |
| *Aspergillus* sp. | ZYJHYZ77 | MT312901 |
| *Aspergillus* sp. | ZYJHYZ180 | MT312962 |
| *Aspergillus* sp. | ZYJHYZ183 | MT312964 |
| *Aspergillus* sp. | ZYJHYZ14 | MT312869 |
| *Aspergillus* sp. | ZYJHYZ92 | MT312911 |
| *Aspergillus* sp. | ZYJHYZ110 | MT312922 |
| *Aspergillus* sp. | ZYJHYZ195 | MT312967 |
| *Aspergillus* sp. | ZYJHYZ221 | MT312970 |
| *Aspergillus* sp. | ZYJHYZ241 | MW659117 |
| *Aspergillus* sp. | ZYJHYZ260 | MW659134 |
| *Aspergillus* sp. | ZYJHYZ12 | MT312868 |
| *Aspergillus* sp. | ZYJHYZ15 | MT312870 |
| *Aspergillus* sp. | ZYJHYZ23 | MT312873 |
| *Aspergillus* sp. | ZYJHYZ24 | MT312874 |
| *Aspergillus* sp. | ZYJHYZ38 | MT312880 |
| *Aspergillus* sp. | ZYJHYZ58 | MT312890 |
| *Aspergillus* sp. | ZYJHYZ71 | MT312896 |
| *Aspergillus* sp. | ZYJHYZ82 | MT312904 |
| *Aspergillus* sp. | ZYJHYZ11 | MT312867 |
| *Aspergillus* sp. | ZYJHYZ26 | MT312875 |
| *Aspergillus* sp. | ZYJHYZ32 | MT312877 |
| *Aspergillus* sp. | ZYJHYZ41 | MT312883 |
| *Aspergillus* sp. | ZYJHYZ47 | MT312886 |
| *Aspergillus* sp. | ZYJHYZ57 | MT312889 |
| *Aspergillus* sp. | ZYJHYZ74 | MT312898 |
| *Aspergillus* sp. | ZYJHYZ84 | MT312905 |
| *Aspergillus* sp. | ZYJHYZ88 | MT312907 |
| *Aspergillus* sp. | ZYJHYZ90 | MT312909 |
| *Aspergillus* sp. | ZYJHYZ91 | MT312910 |
| *Aspergillus* sp. | ZYJHYZ108 | MT312920 |
| *Aspergillus* sp. | ZYJHYZ115 | MT312925 |
| *Aspergillus* sp. | ZYJHYZ116 | MT312926 |
| *Aspergillus* sp. | ZYJHYZ133 | MT312934 |
| *Aspergillus* sp. | ZYJHYZ139 | MT312938 |
| *Aspergillus* sp. | ZYJHYZ143 | MT312940 |
| *Aspergillus* sp. | ZYJHYZ145 | MT312941 |
| *Aspergillus* sp. | ZYJHYZ152 | MT312944 |
| *Aspergillus* sp. | ZYJHYZ160 | MT312949 |
| *Aspergillus* sp. | ZYJHYZ172 | MT312955 |
| *Aspergillus* sp. | ZYJHYZ184 | MT312965 |
| *Aspergillus* sp. | ZYJHYZ193 | MT312966 |
| *Aspergillus* sp. | ZYJHYZ212 | MT312969 |
| *Aspergillus* sp. | ZYJHYZ236 | MW494677 |
| *Aspergillus* sp. | ZYJHYZ238 | MW659114 |
| *Aspergillus* sp. | ZYJHYZ266 | MW659140 |
| *Aspergillus* sp. | ZYJHYZ279 | MW659151 |
| *Aspergillus* sp. | ZYJHYZ03 | MT312866 |
| *Aspergillus* sp. | ZYJHYZ18 | MT312871 |
| *Aspergillus* sp. | ZYJHYZ21 | MT312872 |
| *Aspergillus* sp. | ZYJHYZ35 | MT312878 |
| *Aspergillus* sp. | ZYJHYZ37 | MT312879 |
| *Aspergillus* sp. | ZYJHYZ39 | MT312881 |
| *Aspergillus* sp. | ZYJHYZ40 | MT312882 |
| *Aspergillus* sp. | ZYJHYZ44 | MT312884 |
| *Aspergillus* sp. | ZYJHYZ49 | MT312887 |
| *Aspergillus* sp. | ZYJHYZ60 | MT312891 |
| *Aspergillus* sp. | ZYJHYZ63 | MT312892 |
| *Aspergillus* sp. | ZYJHYZ65 | MT312893 |
| *Aspergillus* sp. | ZYJHYZ69 | MT312894 |
| *Aspergillus* sp. | ZYJHYZ70 | MT312895 |
| *Aspergillus* sp. | ZYJHYZ72 | MT312897 |
| *Aspergillus* sp. | ZYJHYZ75 | MT312899 |
| *Aspergillus* sp. | ZYJHYZ76 | MT312900 |
| *Aspergillus* sp. | ZYJHYZ78 | MT312902 |
| *Aspergillus* sp. | ZYJHYZ85 | MT312906 |
| *Aspergillus* sp. | ZYJHYZ89 | MT312908 |
| *Aspergillus* sp. | ZYJHYZ95 | MT312912 |
| *Aspergillus* sp. | ZYJHYZ100 | MT312913 |
| *Aspergillus* sp. | ZYJHYZ102 | MT312915 |
| *Aspergillus* sp. | ZYJHYZ103 | MT312916 |
| *Aspergillus* sp. | ZYJHYZ104 | MT312917 |
| *Aspergillus* sp. | ZYJHYZ105 | MT312918 |
| *Aspergillus* sp. | ZYJHYZ106 | MT312919 |
| *Aspergillus* sp. | ZYJHYZ107 | MT312920 |
| *Aspergillus* sp. | ZYJHYZ111 | MT312923 |
| *Aspergillus* sp. | ZYJHYZ114 | MT312924 |
| *Aspergillus* sp. | ZYJHYZ125 | MT312928 |
| *Aspergillus* sp. | ZYJHYZ126 | MT312929 |
| *Aspergillus* sp. | ZYJHYZ127 | MT312930 |
| *Aspergillus* sp. | ZYJHYZ128 | MT312931 |
| *Aspergillus* sp. | ZYJHYZ131 | MT312932 |
| *Aspergillus* sp. | ZYJHYZ132 | MT312933 |
| *Aspergillus* sp. | ZYJHYZ136 | MT312935 |
| *Aspergillus* sp. | ZYJHYZ137 | MT312936 |
| *Aspergillus* sp. | ZYJHYZ138 | MT312937 |
| *Aspergillus* sp. | ZYJHYZ141 | MT312939 |
| *Aspergillus* sp. | ZYJHYZ146 | MT312942 |
| *Aspergillus* sp. | ZYJHYZ149 | MT312943 |
| *Aspergillus* sp. | ZYJHYZ154 | MT312945 |
| *Aspergillus* sp. | ZYJHYZ156 | MT312946 |
| *Aspergillus* sp. | ZYJHYZ157 | MT312947 |
| *Aspergillus* sp. | ZYJHYZ158 | MT312948 |
| *Aspergillus* sp. | ZYJHYZ167 | MT312951 |
| *Aspergillus* sp. | ZYJHYZ168 | MT312952 |
| *Aspergillus* sp. | ZYJHYZ169 | MT312953 |
| *Aspergillus* sp. | ZYJHYZ170 | MT312954 |
| *Aspergillus* sp. | ZYJHYZ174 | MT312957 |
| *Aspergillus* sp. | ZYJHYZ175 | MT312958 |
| *Aspergillus* sp. | ZYJHYZ176 | MT312959 |
| *Aspergillus* sp. | ZYJHYZ177 | MT312960 |
| *Aspergillus* sp. | ZYJHYZ178 | MT312961 |
| *Aspergillus* sp. | ZYJHYZ181 | MT312963 |
| *Aspergillus* sp. | ZYJHYZ198 | MT312968 |
| *Aspergillus* sp. | ZYJHYZ224 | MT312971 |
| *Aspergillus* sp. | ZYJHYZ226 | MT312972 |
| *Aspergillus* sp. | ZYJHYZ227 | MT312973 |
| *Aspergillus* sp. | ZYJHYZ230 | MT312974 |
| *Aspergillus* sp. | ZYJHYZ232 | MT312975 |
| *Aspergillus* sp. | ZYJHYZ233 | MT312976 |
| *Aspergillus* sp. | ZYJHYZ242 | MW659118 |
| *Aspergillus* sp. | ZYJHYZ278 | MW659150 |
| *Aspergillus* sp. | ZYJHYZ264 | MW659138 |
| *Aspergillus* sp. | ZYJHYZ80 | MT312903 |
| *Aspergillus* sp. | ZYJHYZ101 | MT312914 |
| *Aspergillus* sp. | ZYJHYZ121 | MT312927 |
| *Aspergillus* sp. | ZYJHYZ166 | MT312950 |
| *Aspergillus* sp. | ZYJHYZ173 | MT312956 |
| *Bjerkandera* sp. | ZYJHYZ255 | MW659130 |
| *Bjerkandera* sp. | ZYJHYZ257 | MW659131 |
| *Bjerkandera* sp. | ZYJHYZ262 | MW659136 |
| *Bjerkandera* sp. | ZYJHYZ273 | MW659145 |
| *Bjerkandera* sp. | ZYJHYZ286 | MZ321983 |
| *Bjerkandera* sp. | ZYJHYZ291 | MZ321988 |
| *Bjerkandera* sp. | ZYJHYZ292 | MZ321989 |
| *Bjerkandera* sp. | ZYJHYZ293 | MZ321990 |
| *Bjerkandera* sp. | ZYJHYZ294 | MZ321991 |
| *Bjerkandera* sp. | ZYJHYZ297 | MZ321994 |
| *Bjerkandera* sp. | ZYJHYZ298 | MZ321995 |
| *Candida* sp. | ZYJHYZ147 | MT313085 |
| *Candida* sp. | ZYJHYZ188 | MT313084 |
| *Candida* sp. | ZYJHYZ189 | MT313083 |
| *Candida* sp. | ZYJHYZ205 | MT313082 |
| *Candida* sp. | ZYJHYZ216 | MT313081 |
| *Cladosporium* sp. | ZYJHYZ265 | MW659139 |
| *Cladosporium* sp. | ZYJHYZ270 | MW659142 |
| *Coniochaeta* sp. | ZYJHYZ164 | MT313010 |
| *Coniochaeta* sp. | ZYJHYZ186 | MT313023 |
| *Coniochaeta* sp. | ZYJHYZ191 | MT313012 |
| *Coniochaeta* sp. | ZYJHYZ213 | MT313024 |
| *Coniochaeta* sp. | ZYJHYZ215 | MT313025 |
| *Coniochaeta* sp. | ZYJHYZ223 | MT313019 |
| *Coniochaeta* sp. | ZYJHYZ234 | MT313021 |
| *Coniochaeta* sp. | ZYJHYZ19 | MT313007 |
| *Coniochaeta* sp. | ZYJHYZ113 | MT313018 |
| *Coniochaeta* sp. | ZYJHYZ155 | MT313008 |
| *Coniochaeta* sp. | ZYJHYZ162 | MT313009 |
| *Coniochaeta* sp. | ZYJHYZ182 | MT313011 |
| *Coniochaeta* sp. | ZYJHYZ199 | MT313013 |
| *Coniochaeta* sp. | ZYJHYZ201 | MT313014 |
| *Coniochaeta* sp. | ZYJHYZ202 | MT313015 |
| *Coniochaeta* sp. | ZYJHYZ203 | MT313016 |
| *Coniochaeta* sp. | ZYJHYZ206 | MT313017 |
| *Coniochaeta* sp. | ZYJHYZ229 | MT313020 |
| *Coniochaeta* sp. | ZYJHYZ235 | MT313022 |
| *Coniochaeta* sp. | ZYJHYZ250 | MW659126 |
| *Coniochaeta* sp. | ZYJHYZ244 | MW659120 |
| *Coniochaeta* sp. | ZYJHYZ246 | MW659122 |
| *Coniochaeta* sp. | ZYJHYZ248 | MW659124 |
| *Crinipellis* sp. | ZYJHYZ17 | MT313068 |
| *Crinipellis* sp. | ZYJHYZ34 | MT313066 |
| *Crinipellis* sp. | ZYJHYZ48 | MT313069 |
| *Crinipellis* sp. | ZYJHYZ59 | MT313067 |
| *Crinipellis* sp. | ZYJHYZ161 | MT313065 |
| *Cryptomarasmius* sp. | ZYJHYZ267 | MW659159 |
| *Cryptomarasmius* sp. | ZYJHYZ284 | MW659156 |
| *Daedaleopsis* sp. | ZYJHYZ282 | MW659154 |
| *Daldinia* sp. | ZYJHYZ04 | MT313070 |
| *Daldinia* sp. | ZYJHYZ50 | MT313071 |
| *Daldinia* sp. | ZYJHYZ62 | MT313072 |
| *Daldinia* sp. | ZYJHYZ99 | MT313074 |
| *Daldinia* sp. | ZYJHYZ192 | MT313073 |
| *Epicoccum* sp. | ZYJHYZ274 | MW659146 |
| *Filobasidium* sp. | ZYJHYZ08 | MT313026 |
| *Filobasidium* sp. | ZYJHYZ13 | MT313027 |
| *Filobasidium* sp. | ZYJHYZ20 | MT313028 |
| *Filobasidium* sp. | ZYJHYZ25 | MT313029 |
| *Filobasidium* sp. | ZYJHYZ29 | MT313030 |
| *Filobasidium* sp. | ZYJHYZ64 | MT313031 |
| *Filobasidium* sp. | ZYJHYZ66 | MT313032 |
| *Filobasidium* sp. | ZYJHYZ67 | MT313033 |
| *Filobasidium* sp. | ZYJHYZ79 | MT313034 |
| *Filobasidium* sp. | ZYJHYZ129 | MT313035 |
| *Filobasidium* sp. | ZYJHYZ140 | MT313036 |
| *Filobasidium* sp. | ZYJHYZ142 | MT313037 |
| *Filobasidium* sp. | ZYJHYZ144 | MT313038 |
| *Filobasidium* sp. | ZYJHYZ150 | MT313039 |
| *Filobasidium* sp. | ZYJHYZ151 | MT313040 |
| *Filobasidium* sp. | ZYJHYZ159 | MT313041 |
| *Filobasidium* sp. | ZYJHYZ171 | MT313042 |
| *Filobasidium* sp. | ZYJHYZ190 | MT313043 |
| *Filobasidium* sp. | ZYJHYZ211 | MT313044 |
| *Fomitopsis* sp. | ZYJHYZ247 | MW659123 |
| *Isaria* sp. | ZYJHYZ240 | MW659116 |
| *Isaria* sp. | ZYJHYZ259 | MW659133 |
| *Isaria* sp. | ZYJHYZ285 | MZ321982 |
| *Isaria* sp. | ZYJHYZ287 | MZ321984 |
| *Isaria* sp. | ZYJHYZ299 | MZ321996 |
| *Meyerozyma* sp. | ZYJHYZ07 | MT313086 |
| *Meyerozyma* sp. | ZYJHYZ27 | MT313087 |
| *Meyerozyma* sp. | ZYJHYZ42 | MT313088 |
| *Meyerozyma* sp. | ZYJHYZ51 | MT313089 |
| *Meyerozyma* sp. | ZYJHYZ96 | MT313090 |
| *Meyerozyma* sp. | ZYJHYZ97 | MT313091 |
| *Meyerozyma* sp. | ZYJHYZ98 | MT313092 |
| *Meyerozyma* sp. | ZYJHYZ119 | MT313093 |
| *Meyerozyma* sp. | ZYJHYZ120 | MT313094 |
| *Meyerozyma* sp. | ZYJHYZ122 | MT313095 |
| *Meyerozyma* sp. | ZYJHYZ123 | MT313096 |
| *Meyerozyma* sp. | ZYJHYZ124 | MT313097 |
| *Meyerozyma* sp. | ZYJHYZ194 | MT313098 |
| *Meyerozyma* sp. | ZYJHYZ214 | MT313099 |
| *Mucor* sp. | ZYJHYZ53 | MT313061 |
| *Mucor* sp. | ZYJHYZ86 | MT313064 |
| *Mucor* sp. | ZYJHYZ87 | MT313060 |
| *Mucor* sp. | ZYJHYZ94 | MT313062 |
| *Mucor* sp. | ZYJHYZ109 | MT313063 |
| *Mucor* sp. | ZYJHYZ185 | MT313056 |
| *Mucor* sp. | ZYJHYZ196 | MT313059 |
| *Mucor* sp. | ZYJHYZ208 | MT313055 |
| *Mucor* sp. | ZYJHYZ217 | MT313052 |
| *Mucor* sp. | ZYJHYZ218 | MT313053 |
| *Mucor* sp. | ZYJHYZ219 | MT313054 |
| *Mucor* sp. | ZYJHYZ220 | MT313057 |
| *Mucor* sp. | ZYJHYZ231 | MT313058 |
| *Mucor* sp. | ZYJHYZ237 | MW659113 |
| *Mucor* sp. | ZYJHYZ239 | MW659115 |
| *Mucor* sp. | ZYJHYZ258 | MW659132 |
| *Nemania* sp. | ZYJHYZ263 | MW659137 |
| *Neopestalotiopsis* sp. | ZYJHYZ269 | MW659141 |
| *Paraconiothyrium* sp. | ZYJHYZ243 | MW659119 |
| *Paraconiothyrium* sp. | ZYJHYZ245 | MW659121 |
| *Paraconiothyrium* sp. | ZYJHYZ249 | MW659125 |
| *Penicillium* sp. | ZYJHYZ01 | MT312977 |
| *Penicillium* sp. | ZYJHYZ06 | MT312979 |
| *Penicillium* sp. | ZYJHYZ10 | MT312980 |
| *Penicillium* sp. | ZYJHYZ16 | MT312981 |
| *Penicillium* sp. | ZYJHYZ33 | MT312983 |
| *Penicillium* sp. | ZYJHYZ45 | MT312984 |
| *Penicillium* sp. | ZYJHYZ55 | MT312986 |
| *Penicillium* sp. | ZYJHYZ61 | MT312987 |
| *Penicillium* sp. | ZYJHYZ209 | MT312993 |
| *Penicillium* sp. | ZYJHYZ05 | MT312978 |
| *Penicillium* sp. | ZYJHYZ30 | MT312982 |
| *Penicillium* sp. | ZYJHYZ54 | MT312985 |
| *Penicillium* sp. | ZYJHYZ68 | MT312988 |
| *Penicillium* sp. | ZYJHYZ83 | MT312989 |
| *Penicillium* sp. | ZYJHYZ134 | MT312990 |
| *Penicillium* sp. | ZYJHYZ135 | MT312991 |
| *Penicillium* sp. | ZYJHYZ165 | MT312992 |
| *Penicillium* sp. | ZYJHYZ271 | MW659143 |
| *Pestalotiopsis* sp. | ZYJHYZ272 | MW659144 |
| *Phaeophlebiopsis* sp. | ZYJHYZ254 | MW659157 |
| *Phaeophlebiopsis* sp. | ZYJHYZ280 | MW659152 |
| *Phaeophlebiopsis* sp. | ZYJHYZ300 | MZ321997 |
| *Piloderma* sp. | ZYJHYZ268 | MW659160 |
| *Piloderma* sp. | ZYJHYZ296 | MZ321993 |
| *Rhizomucor* sp. | ZYJHYZ81 | MT313001 |
| *Rhizomucor* sp. | ZYJHYZ93 | MT313002 |
| *Rhizomucor* sp. | ZYJHYZ112 | MT313004 |
| *Rhizomucor* sp. | ZYJHYZ118 | MT313003 |
| *Rhizomucor* sp. | ZYJHYZ197 | MT313000 |
| *Rhizomucor* sp. | ZYJHYZ222 | MT312999 |
| *Rhizomucor* sp. | ZYJHYZ225 | MT313005 |
| *Rhizomucor* sp. | ZYJHYZ228 | MT313006 |
| *Rhodotorula* sp. | ZYJHYZ22 | MT313045 |
| *Rhodotorula* sp. | ZYJHYZ36 | MT313046 |
| *Rhodotorula* sp. | ZYJHYZ73 | MT313047 |
| *Rhodotorula* sp. | ZYJHYZ179 | MT313048 |
| *Rhodotorula* sp. | ZYJHYZ187 | MT313049 |
| *Rhodotorula* sp. | ZYJHYZ200 | MT313050 |
| *Rhodotorula* sp. | ZYJHYZ204 | MT313051 |
| *Steccherinum* sp. | ZYJHYZ256 | MW659158 |
| *Stereum* sp. | ZYJHYZ281 | MW659153 |
| *Strelitziana* sp. | ZYJHYZ251 | MW659127 |
| *Talaromyces* sp. | ZYJHYZ252 | MW659128 |
| *Talaromyces* sp. | ZYJHYZ09 | MT312994 |
| *Talaromyces* sp. | ZYJHYZ28 | MT312995 |
| *Talaromyces* sp. | ZYJHYZ43 | MT312996 |
| *Talaromyces* sp. | ZYJHYZ52 | MT312997 |
| *Talaromyces* sp. | ZYJHYZ163 | MT312998 |
| *Talaromyces* sp. | ZYJHYZ261 | MW659135 |
| *Talaromyces* sp. | ZYJHYZ277 | MW659149 |
| *Talaromyces* sp. | ZYJHYZ288 | MZ321985 |
| *Talaromyces* sp. | ZYJHYZ289 | MZ321986 |
| *Talaromyces* sp. | ZYJHYZ290 | MZ321987 |
| *Talaromyces* sp. | ZYJHYZ295 | MZ321992 |
| *Trametes* sp. | ZYJHYZ253 | MW659129 |
| *Trametes* sp. | ZYJHYZ117 | MT313076 |
| *Trametes* sp. | ZYJHYZ130 | MT313075 |
| *Trametes* sp. | ZYJHYZ148 | MT313077 |
| *Trametes* sp. | ZYJHYZ153 | MT313080 |
| *Trametes* sp. | ZYJHYZ207 | MT313078 |
| *Trametes* sp. | ZYJHYZ210 | MT313079 |
