# Supplementary material for: Potential Fungi Isolated From Anti-biodegradable Chinese Medicine Residue to Degrade Lignocellulose
Source: Front Microbiol. 2022 May 10;13:877884. doi: 10.3389/fmicb.2022.877884 (PMC9127797; doi:10.3389/fmicb.2022.877884)
Supplement: Supplementary file 3 [file Table_2.PDF]

**Table S2 Growth and enzymatic activity comparison of selected fungi grown on solid medium containing cellulose, xylan or aniline blue**

| Strain number | species name | Diameter (D/d) | | | Total ratio | Hydrolysis activities on fifth day |
|---|---|---|---|---|---|---|
| | | CMC-red | Xyl-red | PDA-blue | | |
| Negative control | | | | | | 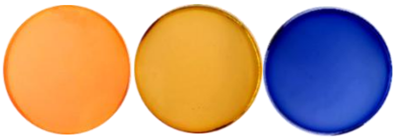 |
| ZYJHYZ257 | *Bjerkandera* sp. | 1 | 1.5 | 1 | 3.5 | 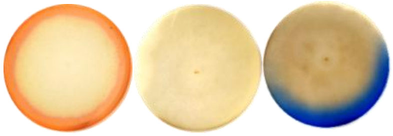 |
| ZYJHYZ258 | *Mucor* sp. | 1 | - | - | 1 | 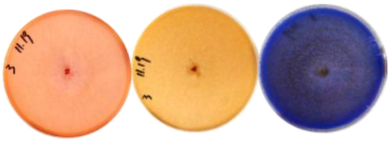 |
| ZYJHYZ259 | *Isaria* sp. | 1.5 | - | 1 | 2.5 | 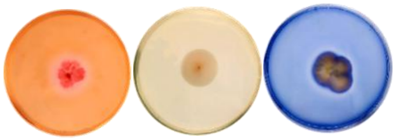 |
| ZYJHYZ260 | *Aspergillus* sp. | 1 | 1 | - | 2 | 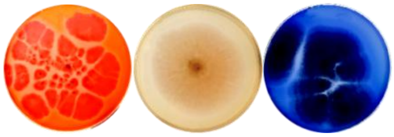 |
| ZYJHYZ261 | *Talaromyces* sp. | - | 1 | 1 | 2 | 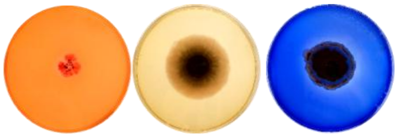 |
| ZYJHYZ262 | *Bjerkandera* sp. | 1 | - | - | 1 | 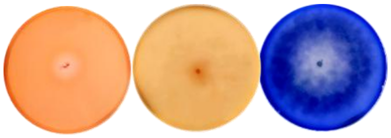 |
| ZYJHYZ263 | *Nemania* sp. | 1 | 1 | 1 | 3 | 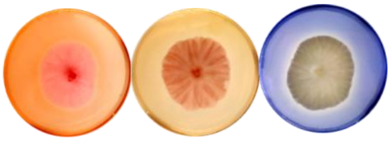 |

| | | | | | | |
|---|---|---|---|---|---|---|
| ZYJHYZ264 | *Aspergillus* sp. | 1 | - | - | 1 | 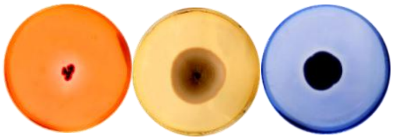 |
| ZYJHYZ265 | *Cladosporium* sp. | 1 | 1 | 1.5 | 3.5 | 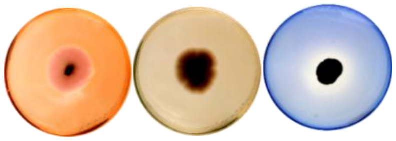 |
| ZYJHYZ267 | *Cryptomarasmius* sp. | 1 | - | 1 | 1 | 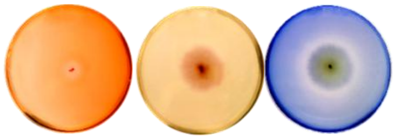 |
| ZYJHYZ268 | *Piloderma* sp. | 1 | 1 | - | 2 | 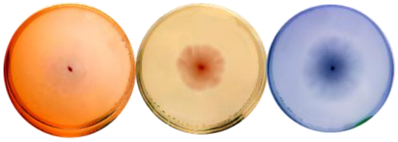 |
| ZYJHYZ269 | *Neopestalotiopsis* sp. | 1 | 1 | 1 | 3 | 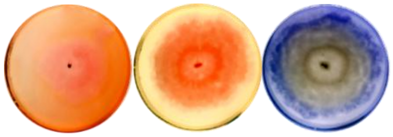 |
| ZYJHYZ270 | *Cladosporium* sp. | 1 | 1 | 1.5 | 3.5 | 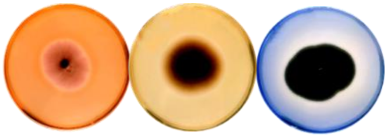 |
| ZYJHYZ271 | *Penicillium* sp. | 1 | 1 | - | 2 | 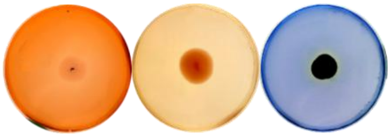 |
| ZYJHYZ296 | *Piloderma* sp. | - | 1 | - | 1 | 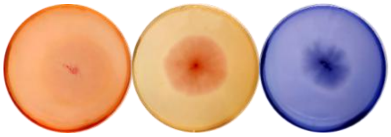 |
| ZYJHYZ272 | *Pestalotiopsis* sp. | 1 | 1 | - | 2 | 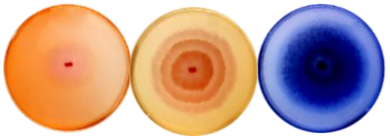 |
| ZYJHYZ273 | *Bjerkandera* sp. | 1 | 1 | 1 | 3 | 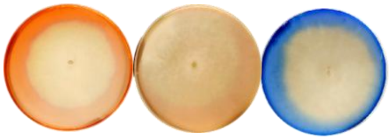 |

| | | | | | | |
|---|---|---|---|---|---|---|
| ZYJHYZ274 | *Epicoccum* sp. | - | 1 | 1 | 2 | 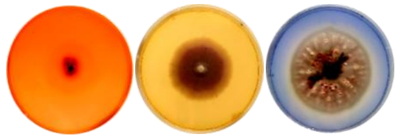 |
| ZYJHYZ278 | *Aspergillus* sp. | 1 | - | - | 1 | 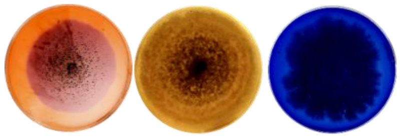 |
| ZYJHYZ279 | *Aspergillus* sp. | 1 | 1 | - | 2 | 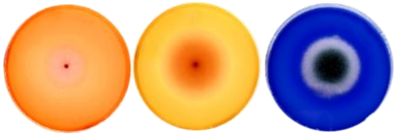 |
| ZYJHYZ184 | *Aspergillus* sp. | 1 | - | - | 1 | 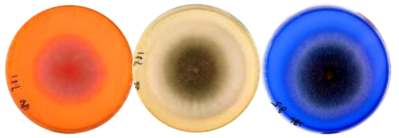 |
| ZYJHYZ221 | *Aspergillus* sp. | 1 | - | - | 1 | 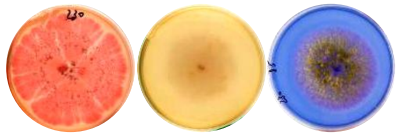 |
| ZYJHYZ163 | *Talaromyces* sp. | 1.5 | 1 | 1 | 3.5 | 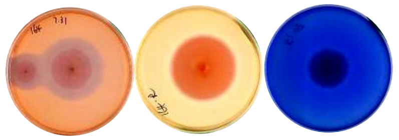 |
| ZYJHYZ53 | *Mucor* sp. | 1 | - | 1 | 2 | 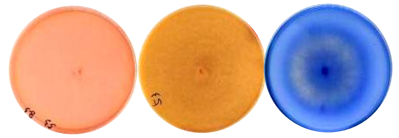 |
| ZYJHYZ190 | *Aspergillus* sp. | 1 | - | - | 1 | 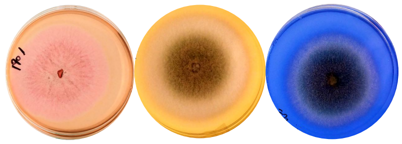 |
| ZYJHYZ244 | *Coniochaeta* sp. | 1 | - | 2 | 3 | 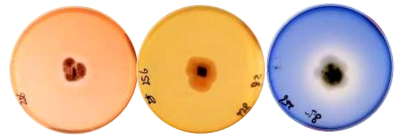 |
| ZYJHYZ242 | *Aspergillus* sp. | 1.5 | 1 | - | 2.5 | 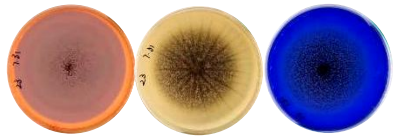 |

| | | | | | | |
|---|---|---|---|---|---|---|
| ZYJHYZ28 | *Talaromyces* sp. | 1 | 1 | 1 | 3 | 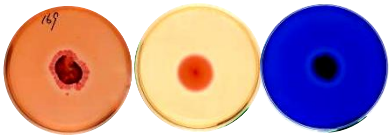 |
| ZYJHYZ246 | *Coniochaeta* sp. | 1 | - | 2 | 3 | 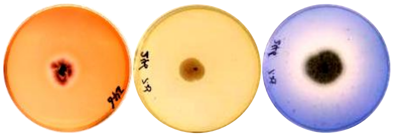 |
| ZYJHYZ236 | *Aspergillus* sp. | 1 | - | - | 1 | 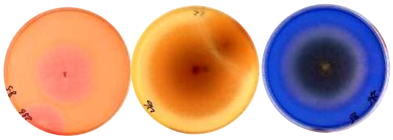 |
| ZYJHYZ254 | *Phaeophlebiopsis* sp. | 1.5 | 1 | 1 | 3.5 | 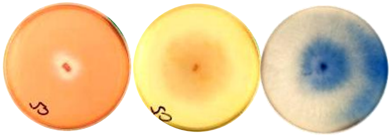 |
| ZYJHYZ255 | *Bjerkandera* sp. | 1 | 1 | 1 | 3 | 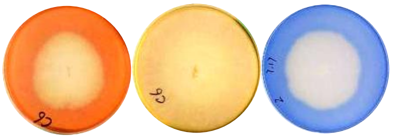 |
| ZYJHYZ240 | *Isaria* sp. | 1 | - | 1.5 | 2.5 | 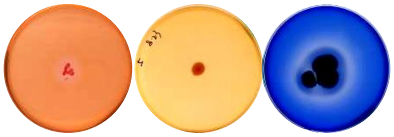 |
| ZYJHYZ247 | *Fomitopsis* sp. | 1 | 1 | - | 2 | 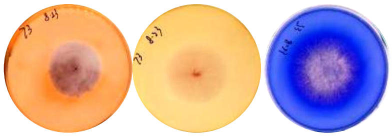 |
| ZYJHYZ256 | *Steccherinum* sp. | 1 | 1 | 1 | 3 | 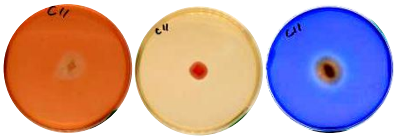 |
