# Supplementary material for: Potential Fungi Isolated From Anti-biodegradable Chinese Medicine Residue to Degrade Lignocellulose
Source: Front Microbiol. 2022 May 10;13:877884. doi: 10.3389/fmicb.2022.877884 (PMC9127797; doi:10.3389/fmicb.2022.877884)
Supplement: Supplementary file 4 [file Table_3.DOC]

**Table S3 The BLAST results from NCBI of the first 3 fungal strains with highest activity of each enzyme**

| Strain number | Genus name | GenBank accession number | BLAST search results | | | | |
| --- | --- | --- | --- | --- | --- | --- | --- |
|  |  |  | Closest match | GenBank accession number | Identity | Query coverage | Reference |
| ZYJHYZ242 | *Aspergillus* sp. | MW659118 | *Aspergillus niger* | EF661186 | 100% | 100% | (Vesth et al., 2018) |
|  |  |  | *Aspergillus niger* | FJ629337 | 100% | 100% | (Hu and Wright, 2021) |
|  |  |  | *Aspergillus niger* | MH862766 | 100% | 100% | (Vu et al., 2019) |
|  |  |  | *Aspergillus niger* | NR111348 | 100% | 98% | (Schoch et al., 2014) |
| ZYJHYZ247 | *Fomitopsis* sp. | MW659123 | *Fomitopsis* *palustris* | MH858571 | 99.17% | 99% | (Vu et al., 2019) |
|  |  |  | *Fomitopsis palustris* | MH855567 | 98.85% | 100% | (Vu et al., 2019) |
|  |  |  | *Fomitopsis betulina* | MH856908 | 91.08% | 95% | (Vu et al., 2019) |
|  |  |  | *Fomitopsis cajanderi* | MH855989 | 88.21% | 100% | (Vu et al., 2019) |
| ZYJHYZ263 | *Nemania* sp. | MW659137 | *Nemania diffusa* | KY555020 | 99.13% | 96% | (Park et al., 2017) |
|  |  |  | *Nemania diffusa* | MZ724900 | 99.12% | 94% | (Zhang et al., 2021) |
|  |  |  | *Nemania diffusa* | MT509577 | 96.04% | 92% | (Yan et al., 2021) |
|  |  |  | *Nemania diffusa* | MF185340 | 97.06% | 96% | (María et al., 2018) |
| ZYJHYZ163 | *Talaromyces* sp. | MT312998 | *Talaromyces purpureogenus* | MH865647 | 100% | 100% | (Vu et al., 2019) |
|  |  |  | *Talaromyces purpureogenus* | MH854924 | 99.8% | 100% | (Vu et al., 2019) |
|  |  |  | *Talaromyces purpureogenus* | MH865216 | 96.67% | 100% | (Vu et al., 2019) |
|  |  |  | *Talaromyces purpureogenus* | HQ608123 | 91.29% | 100% | (Rodrigues et al., 2011) |
| ZYJHYZ254 | *Phaeophlebiopsis* sp. | MW659157 | *Phaeophlebiopsis* sp. | KP135412 | 87.7% | 100% | (Floudas and Hibbett, 2015) |
|  |  |  | *Phaeophlebiopsis* sp. | KP135413 | 87.13% | 97% | (Floudas and Hibbett, 2015) |
|  |  |  | *Phaeophlebiopsis ravenelii* | MH856691 | 87.45% | 100% | (Vu et al., 2019) |
|  |  |  | *Phaeophlebiopsis* sp. | MZ637016 | 86.85% | 92% | (Chen et al., 2021) |
| ZYJHYZ257 | *Bjerkandera* sp. | MW659131 | *Bjerkandera adusta* | GU067733 | 98.95% | 100% | (Vasaitis et al., 2016) |
|  |  |  | *Bjerkandera adusta* | MH857085 | 99.46% | 97% | (Vu et al., 2019) |
|  |  |  | *Bjerkandera adusta* | EF441742 | 99.06% | 93% | (Romero et al., 2007) |
|  |  |  | *Bjerkandera adusta* | MH856704 | 98.19% | 97% | (Vu et al., 2019) |
| ZYJHYZ246 | *Coniochaeta* sp. | MW659122 | *Coniochaeta velutina* | JQ346221 | 100% | 100% | (Vaz et al., 2014) |
|  |  |  | *Coniochaeta velutina* | MH859264 | 99.81% | 100% | (Vu et al., 2019) |
|  |  |  | *Coniochaeta velutina* | KP776995 | 100% | 90% | (Xie et al., 2015) |
|  |  |  | *Coniochaeta* *velutina* | MT813993 | 100% | 87% | (Spies et al., 2020) |
| ZYJHYZ268 | *Piloderma* sp. | MW659160 | *Piloderma olivaceum* | MH864982 | 82.62% | 95% | (Vu et al., 2019) |
|  |  |  | *Piloderma fallax* | MH861062 | 82.18% | 95% | (Vu et al., 2019) |
|  |  |  | *Piloderma byssinum* | MH864980 | 80.06% | 95% | (Vu et al., 2019) |

**References**

Chen, C. C., Chen, C. Y., Wu, S. H. (2021). Species diversity,taxonomy and multi-gene phylogeny of phlebioid clade (*Phanerochaetaceae*, irpicaceae, meruliaceae) of polyporales. *Fungal. divers*. 6, 106.

Floudas, D., & Hibbett, D. S. (2015). Revisiting the taxonomy of *Phanerochaete* (*Polyporales*, Basidiomycota) using a four gene dataset and extensive ITS sampling. *Fungal. Biol.* 119, 8, 679-719. [doi: org/10.1016/j.funbio.2015.04.003](https://doi.org/10.1016/j.funbio.2015.04.003)

Hu, J., & Wright, G. (2021). First report of *Fomitopsis meliae* causing brown wood rot on living lemon trees in Arizona and California. *Plant. Dis*. 10.1094. [doi: 10.1094/PDIS-11-20-2427-PDN](https://doi.org/10.1094/PDIS-11-20-2427-PDN)

María, F. D., Chaves, W., Jeniffer Y. (2018). Fungal communities associated with symptomatic leaves of naranjilla (solanum quitoense lam.) in the ecuadorian amazon region. *Rev. Ecuat. Med. Cienc. Biol*. 39, 1, 39-49. doi: 10.26807/remcb.v39i1.561

Park, J. M. , You, Y. H. , Back, C. G. , Kim, H. H. , Ghim, S. Y. , Park, J. H. (2017). Fungal load in bradysia agrestis, a phytopathogen-transmitting insect vector. *Symbiosis*. 74, 145-158. doi: 10.1007/s13199-017-0494-3

Rodrigues, A., Mueller, U. G., Ishak, H. D., Bacci, M., Jr, Pagnocca, F. C. (2011). Ecology of microfungal communities in gardens of fungus-growing ants (*Hymenoptera*: *Formicidae*): a year-long survey of three species of attine ants in Central Texas. *FEMS. Microbiol. Ecol*. 78, 2, 244-255. [doi: org/10.1111/j.1574-6941.2011.01152.x](https://doi.org/10.1111/j.1574-6941.2011.01152.x)

Romero, E., Speranza, M., García-Guinea, J., Martínez, A. T., Martínez, M. J. (2007). An anamorph of the white-rot fungus *Bjerkandera adusta* capable of colonizing and degrading compact disc components.*FEMS. Microbiol. Lett.* 275, 1, 122-129. [doi: org/10.1111/j.1574-6968.2007.00876.x](https://doi.org/10.1111/j.1574-6968.2007.00876.x)

Schoch, C. L., Robbertse, B., Robert, V., Vu, D., Cardinali, G., Irinyi, L., et al. (2014). Finding needles in haystacks: linking scientific names, reference specimens and molecular data for Fungi. *Database. (Oxford)*.bau061. [doi: 10.1093/database/bau061](https://doi.org/10.1093/database/bau061)

Spies, C., Mostert, L., Carlucci, A., Moyo, P., van Jaarsveld, W. J., du Plessis, I. L., et al. (2020). Dieback and decline pathogens of olive trees in South Africa. *Persoonia*. 45, 196-220. doi: org/10.3767/persoonia.2020.45.08

Vasaitis, R., Burnevica, N., Uotila, A., Dahlberg, A., Kasanen, R. (2016). Cut Picea abies stumps constitute low quality substrate for sustaining biodiversity in fungal communities. *Balt. For*. 22, 2: 239-245.

Vaz, A.B.M., Fontenla, S., Rocha, F.S., Brandao, L.R., Vieira, M.L.A., de Garcia,V., et al. (2014). Fungal endophyte-diversity associated with Myrtaceae species in an Andean Patagonian forest (Argentina) and an Atlantic forest (Brazil). *Fungal. Ecol*. 8, 28-36

Vesth, T. C., Nybo, J. L., Theobald, S., Frisvad, J. C., Larsen, T. O., Nielsen, K. F., et al. (2018). Investigation of inter-and intraspecies variation through genome sequencing of *Aspergillus* section Nigri. *Nat. Genet.*50, 12, 1688-1695. [doi: 10.1038/s41588-018-0246-1](https://doi.org/10.1038/s41588-018-0246-1)

Vu, D., Groenewald, M., de Vries, M., Gehrmann, T., Stielow, B., Eberhardt, U., et al. (2019). Large-scale generation and analysis of filamentous fungal DNA barcodes boosts coverage for kingdom fungi and reveals thresholds for fungal species and higher taxon delimitation. *Stud. Mycol.* 92, 135-154. [doi: org/10.1016/j.simyco.2018.05.001](https://doi.org/10.1016/j.simyco.2018.05.001)

Xie, J., Strobel, G. A., Feng, T., Ren, H., Mends, M. T., Zhou, Z., Geary, B. (2015). An endophytic *Coniochaeta velutina* producing broad spectrum antimycotics.  *J. Microbiol*. 53, 6, 390-397. [doi: org/10.1007/s12275-015-5105-5](https://doi.org/10.1007/s12275-015-5105-5)

Yan, J., Is, M., Rs, J., Hl, L., Jy, Y. (2021). Microfungi associated with camellia sinensis: a case study of leaf and shoot necrosis on tea in fujian, China. *Mycosphere*. 12, 1, 430-518. doi: 10.5943/mycosphere/12/1/6

Zhang, H., Wei, T. P., Li, L. Z., Luo, M. Y., Jia, W. Y., Zeng, Y., Jiang, Y. L., Tao, G. C. (2021). Multigene phylogeny, diversity and antimicrobial potential of endophytic *Sordariomycetes* rrom rosa roxburghii. *Front. Microbiol*. 12, 755919. [doi: org/10.3389/fmicb.2021.755919](https://doi.org/10.3389/fmicb.2021.755919)
